# Effects of a Rice Bran Dietary Intervention on the Composition of the Intestinal Microbiota of Adults with a High Risk of Colorectal Cancer: A Pilot Randomised-Controlled Trial

**DOI:** 10.3390/nu13020526

**Published:** 2021-02-06

**Authors:** Winnie K. W. So, Judy Y. W. Chan, Bernard M. H. Law, Kai Chow Choi, Jessica Y. L. Ching, Kam Leung Chan, Raymond S. Y. Tang, Carmen W. H. Chan, Justin C. Y. Wu, Stephen K. W. Tsui

**Affiliations:** 1 The Nethersole School of Nursing, The Chinese University of Hong Kong, Hong Kong, China; judychanyw@cuhk.edu.hk (J.Y.W.C.); bernardlaw@cuhk.edu.hk (B.M.H.L.); kchoi@cuhk.edu.hk (K.C.C.); whchan@cuhk.edu.hk (C.W.H.C.); 2 School of Chinese Medicine, The Chinese University of Hong Kong, Hong Kong, China; jessicaching@cuhk.edu.hk (J.Y.L.C.); chankl@cuhk.edu.hk (K.L.C.); 3 Hong Kong Institute of Integrative Medicine, The Chinese University of Hong Kong, Hong Kong, China; 4 Institute of Digestive Disease, The Chinese University of Hong Kong, Hong Kong, China; raymondtang@cuhk.edu.hk (R.S.Y.T.); justinwu@cuhk.edu.hk (J.C.Y.W.); 5 Department of Medicine and Therapeutics, The Chinese University of Hong Kong, Hong Kong, China; 6 School of Biomedical Sciences, The Chinese University of Hong Kong, Hong Kong, China; kwtsui@cuhk.edu.hk

**Keywords:** rice bran, dietary intervention, intestinal microbiota, intestinal health, colorectal cancer

## Abstract

Rice bran exhibits chemopreventive properties that may help to prevent colorectal cancer (CRC), and a short-term rice bran dietary intervention may promote intestinal health via modification of the intestinal microbiota. We conducted a pilot, double-blind, randomised placebo-controlled trial to assess the feasibility of implementing a long-term (24-week) rice bran dietary intervention in Chinese subjects with a high risk of CRC, and to examine its effects on the composition of their intestinal microbiota. Forty subjects were randomised into the intervention group (*n* = 19) or the control group (*n* = 20). The intervention participants consumed 30 g of rice bran over 24-h intervals for 24 weeks, whilst the control participants consumed 30 g of rice powder on the same schedule. High rates of retention (97.5%) and compliance (≥91.3%) were observed. No adverse effects were reported. The intervention significantly enhanced the intestinal abundance of *Firmicutes* and *Lactobacillus*, and tended to increase the *Firmicutes*/*Bacteroidetes* ratio and the intestinal abundance of *Prevotella_9* and the health-promoting *Lactobacillales* and *Bifidobacteria*, but had no effect on bacterial diversity. Overall, a 24-week rice bran dietary intervention was feasible, and may increase intestinal health by inducing health-promoting modification of the intestinal microbiota. Further larger-scale studies involving a longer intervention duration and multiple follow-up outcome assessments are recommended.

## 1. Introduction

Colorectal cancer (CRC) is one of the most common cancers in humans. Thus, effective chemopreventive strategies against CRC are required, especially for those with a high risk of CRC. Previous chemopreventive strategies relied primarily on pharmacological interventions that involved the use of conventional anti-inflammatory drugs, but their side effects have raised doubts over their safety [1]. Alternative strategies that involve the use of natural products, which may elicit fewer side effects, are needed.

The use of certain natural products in dietary interventions has long been considered a potential strategy for CRC prevention [2]. Indeed, a recent review has also provided multiple lines of evidence for an inverse correlation between the consumption of whole grain foods and CRC risk [3], indicating that dietary interventions involving the consumption of dietary fibre-rich foods may achieve effective CRC prevention. Thus, rice bran, a dietary fibre-rich by-product of rice milling, has emerged as a promising natural product for use in CRC chemopreventive interventions. It serves as an inexpensive and nutritious dietary supplement with the potential to promote intestinal health, as it contains chemopreventive phytochemicals, such as tocotrienols, tocopherols and γ-oryzanol, which have been shown to help alleviate oxidative stress and inflammation [4,5,6], both of which have been implicated in the pathogenesis of CRC. Thus, regular consumption of rice bran may potentially reduce the risk of CRC. In addition, more recent studies have revealed that its chemopreventive effects are due to its health-promoting modulation of the composition of the intestinal microbiota and prevention of microbial dysbiosis [7].

Microbial dysbiosis is a state of imbalance in a bacterial community or microbiota within the body, in which the body’s resident population of pathogenic bacteria outcompete that of health-promoting bacteria. Notably, it has been suggested that intestinal microbial dysbiosis is linked to CRC, because the intestinal abundance of certain bacterial genera was found to be modified in patients with CRC. For example, *Prevotella* were found to be more abundant in colonic mucosal samples obtained from patients with CRC than in those obtained from healthy control subjects [8]. In contrast, the intestinal abundance of *Lactobacillus* and *Bifidobacteria*, two genera of bacteria that are known to promote health, was reduced in these patients [9]. Furthermore, the relative abundance of *Firmicutes* and *Bacteroidetes*, two major bacterial phyla in the gut, was suggested to modify CRC risks in animal studies, as they showed that the formation of colon tumours is associated with a decrease in the *Firmicutes*/*Bacteroidetes* (F/B) ratio [10,11]. Taken together, these findings indicate that maintaining a certain microbiotal composition in the intestines promotes intestinal health, and may be crucial to the prevention of CRC.

Animal studies and randomised controlled trials have provided multiple lines of evidence to show the beneficial effects of rice bran consumption, in terms of enhancing the colonisation of the intestinal microbiota by health-promoting bacteria [12,13,14,15,16,17]. In particular, dietary interventions involving the daily consumption of 30 g of rice bran have been shown to elicit health-promoting changes in the composition of the intestinal microbiota in humans [15,16]. However, it is not clear whether long-term consumption of rice bran by humans would have similar outcomes, as most human studies have involved rice bran dietary interventions that lasted only 4 weeks [15,16,17]. Therefore, a study of a longer-term rice bran dietary intervention is needed to determine whether it results in positive modification of the composition of the intestinal microbiota in the long-term. If so, this would suggest that a rice bran dietary intervention should be used for the chemoprevention of CRC in those who have a high risk of developing this disease. Furthermore, only a few human studies have examined the effects of a rice bran dietary intervention on the intestinal microbiota [15,16,17,18], and only one was conducted in a Chinese population [17]. As the composition of the intestinal microbiota has been shown to differ across ethnicities [19], possibly due to lifestyle and genetic factors that may affect the microbiome composition [20,21], the intake of rice bran is likely to exhibit different effects on the intestinal microbiota of individuals of different ethnicities. Furthermore, although the Chinese study [17] revealed that rice bran intake reduced the F/B ratio of the participants’ intestinal microbiota, its pre-test–post-test design hampered the formulation of firm conclusions on the effects of the intervention on the intestinal microbiota. Thus, a randomised controlled trial of the effects of rice bran intake on the intestinal microbiota of Chinese subjects is needed.

Our study therefore aimed to assess the effects of a 24-week rice bran dietary intervention on the composition of the intestinal microbiota in Chinese adults with a high risk of CRC. Our findings would provide further evidence that rice bran dietary interventions may promote intestinal health, and could be effective for the chemoprevention of CRC.

## 2. Methods

### 2.1. Study Design

This pilot study had a double-blind, two-arm, parallel, randomised placebo-controlled trial design. The trial was registered at the ISRCTN Registry (ISRCTN33245212).

### 2.2. Study Settings and Participants

The sample size for this pilot randomised controlled trial was not determined upon hypothesis testing. However, it was expected to be adequate to examine the feasibility of the intervention and adverse events, and to provide preliminary estimates for the effects of the intervention on intestinal microbiota. According to Lancaster et al. [22], a sample size of 30 participants would generally be adequate for a pilot trial. Allowing for an attrition rate of up to 25%, we sought to recruit 40 eligible participants, and thus have 20 participants in both the intervention group and the control group.

Eligible subjects with a high risk of CRC were recruited via convenient sampling within the community in Hong Kong between January and April 2020. Healthy Chinese adults, who had undergone CRC screening and have agreed to be contacted for future research at the Institute of Digestive Disease, were our potential subjects. Our research staff approached these individuals via phone to screen for their eligibility for participation. Subjects with a high risk of CRC were identified with the use of the scoring system of the Asia-Pacific Colorectal Screening tool, a validated tool that is used to stratify individuals into groups according to their risk of developing CRC, based on factors such as age, gender, smoking habit and family history [23]. The inclusion criteria were (1) self-identification as Chinese and at least 50 years of age; (2) an Asia-Pacific Colorectal Screening score between 4 and 7 (indicating a high risk of CRC); (3) a lack of gastrointestinal symptoms indicative of CRC; (4) a negative result on a faecal occult blood test and/or colonoscopy over the past year; (5) no known history of food allergies or gallstones; (6) no dietary restrictions; (7) no prescription for cholesterol-lowering medications or non-steroidal anti-inflammatory drugs; and (8) no prescription for probiotics, prebiotics, antibiotics or traditional Chinese medicine over the past three months. Subjects were excluded if they had had any gastrointestinal symptoms within the past week, if they were pregnant, undergoing menopause or lactating, or if they had diabetes.

### 2.3. Randomisation and Masking

Block randomisation was used to randomise the eligible subjects into the intervention group or the control group with a 1:1 allocation ratio. Randomisation was conducted with a block size of 10 by an independent statistician using computer-generated random numbers. Allocation concealment was ensured by assigning the group allocation with sequentially numbered sealed opaque envelopes. The subjects and the research nurses involved in subject recruitment and data collection were all blinded to the group allocation.

### 2.4. Intervention

The intervention involved the dietary consumption of rice bran over a 24-week period. With the finding showing the effect of daily consumption of 30 g rice bran on the positive modification of the composition of human intestinal microbiota [15,16], the dosage of rice bran used in this intervention was set to 30 g per day. The intervention participants were given packets of rice bran (Riverdi, Hualien, Taiwan), and were instructed to consume 30 g of rice bran per day, at 24-h intervals, throughout the study period. The participants in the control group were given packets of rice powder (Gerber, Arlington, VA, USA) that had a similar appearance and colour to the rice bran consumed by the intervention participants. The control participants were asked to consume 30 g of the rice powder per day during the study. All participants were instructed not to modify their usual lifestyle practices, including their diet and physical activity levels, during the study period. Each participant was given a container and a measuring scale to enable them to conveniently weigh 30 g of rice bran or rice powder for consumption. To ensure blinding, the packaging labels of the rice bran and rice powder packets were removed before the packets were given to the participants. Table 1 presents the nutritional information of the rice bran and rice powder used in this study.

### 2.5. Outcomes

The primary outcomes of this study were changes in the bacterial diversity, in the intestinal abundance of known health-promoting and pathogenic bacteria and in the F/B ratio of the subjects’ intestinal microbiota before and after the intervention. Bacterial diversity is defined as the richness and evenness of the bacterial community in the intestine, and the F/B ratio is calculated as the ratio between the intestinal abundance of *Firmicutes* and that of *Bacteroidetes* in a subject’s intestinal microbiota. The experimental procedures that were used to assess these outcomes are described in the Procedures section.

The secondary outcomes of the study were the participants’ compliance with the intervention and the occurrence of adverse events associated with rice bran intake, as measured by the participants’ bowel patterns and their experience of any discomfort. To assess compliance, the participants were given a logbook in which they recorded the amount of rice bran (or rice powder) consumed on each day during the intervention, and the time that this was consumed. The compliance of each participant was assessed by calculating the ratio between the number of times the participant consumed the rice bran (or rice powder) and the number of times he or she should have consumed it during the 24-week study (i.e., 168 times). To assess the adverse effects of the intervention, the participants were asked to complete a faecal diary, in which they recorded their frequency of egestion and the amount and form of stool egested each day throughout the 24-week intervention. The report on the form of the egested stool was based on the Bristol Stool Scale classification of stool types [24]. Adverse effects were also assessed by the participants’ self-reported occurrence of abdominal pain or discomfort, diarrhoea, or a bloated feeling.

### 2.6. Data Collection Procedures

Upon receiving written informed consent from the potential subjects, baseline (T0) measurements were taken, and the participants were asked to complete a questionnaire for the collection of demographic information. They were also given the logbook and faecal diary for completion during the study period. The participants were given stool sample containers and sterile collection brushes to collect their stool samples. Follow-up data were collected 24 weeks (T1) after the start of the intervention. Appointments were scheduled for all participants at T1 to deposit their stool samples at the hospital clinic, where the subject recruitment had been conducted.

### 2.7. Procedures

#### 2.7.1. Stool Sample Collection and Processing

At T0 and T1, the participants were asked to collect a stool sample within seven days by transferring 3–4 g of their stool into a preservative buffer-containing collection tube using a sterile collection brush. The participants were asked to store the stool samples at 4 °C until they were collected by data collectors. The samples were transported to the laboratory within 24 h of egestion in containers filled with ice packs and were stored at −80 °C, prior to further processing.

Microbial DNA was extracted from the stool samples using the QIAamp DNA Stool Mini Kit (Qiagen Inc., Germantown, MD, USA) according to the manufacturer’s instructions. The extracted DNA samples were stored at −20 °C, prior to further processing.

#### 2.7.2. 16S Metagenomic Sequencing

The DNA samples were subjected to polymerase chain reaction (PCR) analysis to generate a metagenomic library using the 515F-806R primers that flank the V4 hypervariable region of the 16S ribosomal RNA gene. The sequence of the forward primer is 5′-GTG CCA GCM GCC GCG GTA A-3′, and that of the reverse primer is 5′-GGA CTA CHV HHH TWT CTA AT-3′. PCR was carried out in duplicate for each sample with Phusion High-Fidelity DNA Polymerase (New England Biolabs, Ipswich, MA, USA) under the following conditions: initial denaturation for 1 min at 94 °C, followed by 30 cycles of DNA denaturation for 40 s at 94 °C, primer annealing for 40 s at 50 °C, and primer extension for 1 min at 72 °C. The PCR products were purified with the QIAquick Gel Extraction Kit (Qiagen Inc., Germantown, MD, USA). All PCR products were diluted to 1 to 2 ng/μL and pooled together for next-generation sequencing using the Ion Torrent PGM platform (Invitrogen).

### 2.8. Data Analysis

Appropriate descriptive statistics were used to present the data, which comprised the participants’ baseline characteristics, their compliance with the intervention, any adverse events associated with their rice bran intake, and parameters pertaining to their intestinal bacterial diversity including the number of observed species, the Shannon and beta indices, F/B ratio and the abundance of known health-promoting and pathogenic bacteria. Metagenomic data were analysed with the mothur software package. The METASTATS tool of the mothur software package was used to detect differences in the composition of their intestinal bacterial community at T0 and T1. As the total bacterial counts for each subject were likely to differ from baseline to post-intervention, absolute abundance values were used instead of relative abundance values to report the intestinal abundance of each type of bacteria. The absolute abundance of the top five types of bacteria at each taxonomic level and of bacteria known to be health-promoting or pathogenic was used in the analysis of the effects of rice bran intake on the composition of the intestinal microbiota, and the abundance values were presented as medians and interquartile ranges. The bacterial diversity of the stool microbiota was also examined using the mothur software package, and the inter-group difference in the number of observed species and the Shannon index between T0 and T1 were examined. Linear regression analyses were performed, after appropriate data transformation to correct for skewness, to compare inter-group differences in the extent of changes in outcomes across T0 and T1. Further adjusted outcome comparisons between the intervention and control groups were performed using multiple regression analyses after adjustment for sex. These analyses showed significant differences between the two groups. All statistical analyses were conducted with IBM SPSS 25 (IBM Corp, Armonk, NY, USA). All statistical tests were two-sided, and the level of significance was set at 5%.

### 2.9. Ethical Considerations

Ethical approval was obtained from the joint Chinese University of Hong Kong–New Territories East Hospital Cluster clinical research ethics committee (Reference: 2019.482), and the study was conducted in accordance with the Declaration of Helsinki. Eligible subjects were briefed on the study purpose and procedures involved, using an information sheet. They were informed of their right to withdraw from the study without penalty and of the anonymity and confidentiality of the data provided throughout the study. The participants were enrolled in the study only after they gave written informed consent.

## 3. Results

### 3.1. Subject Recruitment and Retention Rate

**Figure 1:**
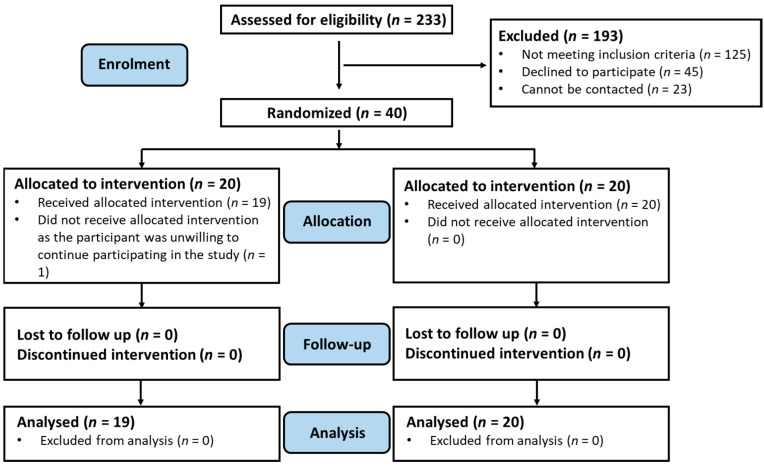
The Consolidated Standards of Reporting Trials (CONSORT) diagram showing the flow of the study.

The flow of the study is depicted in the Consolidated Standards of Reporting Trials diagram (Figure 1). Two hundred and thirty-three subjects were approached by phone for recruitment, of whom 23 could not be reached. The approached subjects were screened for their eligibility for study participation, and 125 were excluded because they did not meet the eligibility criteria. Forty-five eligible subjects declined to participate in the study, primarily due to their reluctance to take the time to participate in a long-term study. Forty subjects were therefore recruited for participation in the study.

Randomisation was carried out for the 40 recruited subjects; 20 subjects were randomised to the intervention group and 20 to the control group. One participant in the intervention group withdrew from the study after randomisation; the subject expressed an unwillingness to continue participating in the study but did not provide further reasons. The study’s retention rate was thus 97.5%. All participants who had provided faecal samples were included in the analysis. Table 2 presents the baseline characteristics of the enrolled participants.

### 3.2. Effect of Rice Bran Intake on Intestinal Microbiota

#### 3.2.1. Composition of Intestinal Microbiota

**Figure 2:**
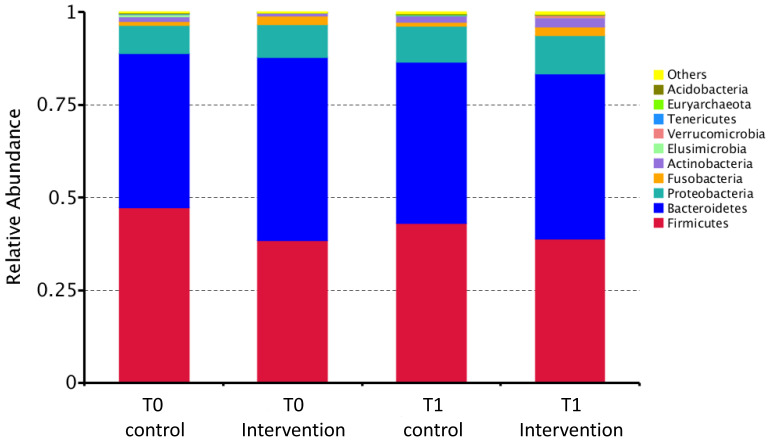
A bar chart showing the relative abundance of the bacteria in the intestinal microbiota at the phylum level among the intervention and control participants at T0 and T1.

Table 3 shows the intestinal abundance of the five most abundant bacteria at each taxonomic level and that of several health-promoting and pathogenic bacterial genera and species that are known to be either enriched or decreased in abundance in the intestinal microbiota of patients with CRC. A graphical representation of the relative intestinal abundance of bacteria at the phylum level in the intervention and control participants at T0 and T1 is shown in Figure 2. Overall, a decrease in the intestinal abundance of *Firmicutes* was observed in the control participants between T0 and T1, and a slight temporal increase in this outcome was observed in their intervention counterparts. The health-promoting bacterial genera and species included *Bifidobacteria*, *Faecalibacterium*, *Blautia*, *Roseburia*, *Ruminococcus*, *Faecalibacterium prausnitzii*, *Bifidobacterium longum,* and *Clostridium butyricum* [9,25]. Pathogenic ones included *Escherichia*–*Shigella*, *Fusobacterium,* and *Bacteroides fragilis* [9,25,26]. The intervention appeared to have an opposite effect on the temporal change in the median absolute abundance of several bacteria at various taxonomic levels, including *Firmicutes*, *Clostridia*, *Clostridiales*, *Bacteroidaceae*, *Lachnospiraceae,* and *Bacteroides*, between the two groups (Table 3).

Table 4 presents the median changes in the absolute abundance values of each bacteria examined between T0 and T1 for both groups. Overall, a greater increase in the absolute abundance values of certain health-promoting bacteria was observed among the intervention participants than among the control subjects. At the phylum level, a significant difference was observed in the change for *Firmicutes* (*p* = 0.018). Moreover, the increase in the intestinal abundance of *Actinobacteria* among the intervention participants at T1 was found to be greater than that among the control counterparts, although the difference did not reach statistical significance (*p* = 0.129). Such a finding is generally reflected in the analysis of bacterial abundance using a heat map (Figure S1), which confirmed that the intestinal abundance of *Firmicutes* was decreased in the control participants between T0 and T1, although no significant effect on this outcome was shown in the intervention participants. Likewise, the intestinal abundance of *Actinobacteria* was more significantly increased among the intervention participants at T1. At the class level, the intervention participants tended to exhibit a greater intestinal abundance of *Bacilli* (*p* = 0.179) than the control participants. At the order level, similar findings were obtained for *Lactobacillales* (*p* = 0.146). At the genus level, a significant increase was seen in the intestinal abundance of *Lactobacillus* among the intervention participants (*p* = 0.033), although the difference in the temporal changes in its intestinal abundance was small. Similar observations were seen for *Bifidobacteria* and *Prevotella_9*, although the inter-group difference in the magnitude of the increase in its intestinal abundance failed to reach statistical significance (*p* = 0.131 and 0.160 respectively). However, no significant inter-group differences were seen in the temporal changes in the intestinal abundance of the aforementioned pathogenic bacteria between T0 and T1 (*p* ≥ 0.647).

Overall, the results showed that consumption of rice bran for 24 weeks may modify the composition of the intestinal microbiota, primarily by increasing the intestinal abundance of certain bacterial genera known to be health-promoting, such as *Lactobacillus* and *Bifidobacteria*. No changes were observed in the abundance of known pathogenic bacteria in the intestinal microbiota.

#### 3.2.2. F/B Ratio

Table 3 shows the median F/B ratios of the intervention and control groups at T0 and T1, and Table 4 shows the median changes in these ratios between T0 and T1. While the F/B ratio of the control participants had decreased at the end of the 24-week intervention, the F/B ratio of the intervention participants showed a slight increase after 24 weeks of rice bran consumption. Nevertheless, the inter-group difference in such changes did not reach statistical significance in this study (*p* = 0.054). The different outcomes observed between the groups at T1 demonstrate that the consumption of rice bran for 24 weeks may increase the F/B ratio in the intestinal microbiota.

#### 3.2.3. Bacterial Diversity

**Figure 3:**
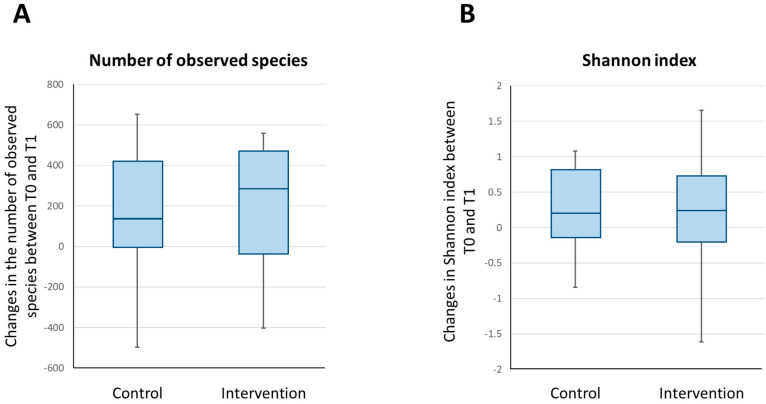
Changes in the alpha diversity parameters of the intestinal microbiota of the participants between baseline and post-intervention. The box and whisker plots show the median, lower quartile, upper quartile, minimum value and maximum value of the number of observed species in the intestinal microbiota of the participants (**A**) and the Shannon index (**B**).

Figure 3 shows a graphical representation of the temporal changes in the number of observed species and the Shannon index for the intervention and control groups. The difference in the change in the number of observed species from T0 to T1 in the intervention group (median change, 289; interquartile range, −38 to 475) and the control group (median change, 133; interquartile range, −4 to 420) was not statistically significant (unadjusted *p* = 0.642 and sex-adjusted *p* = 0.641). Similarly, the difference in the modification of the Shannon index from T0 to T1 in the intervention group (median change, 0.238; interquartile range, −0.199 to 0.722) and the control group (median change, 0.210; interquartile range, −0.136 to 0.828) did not reach statistical significance either (unadjusted *p* = 0.776 and sex-adjusted *p* = 0.566). 

**Figure 4:**
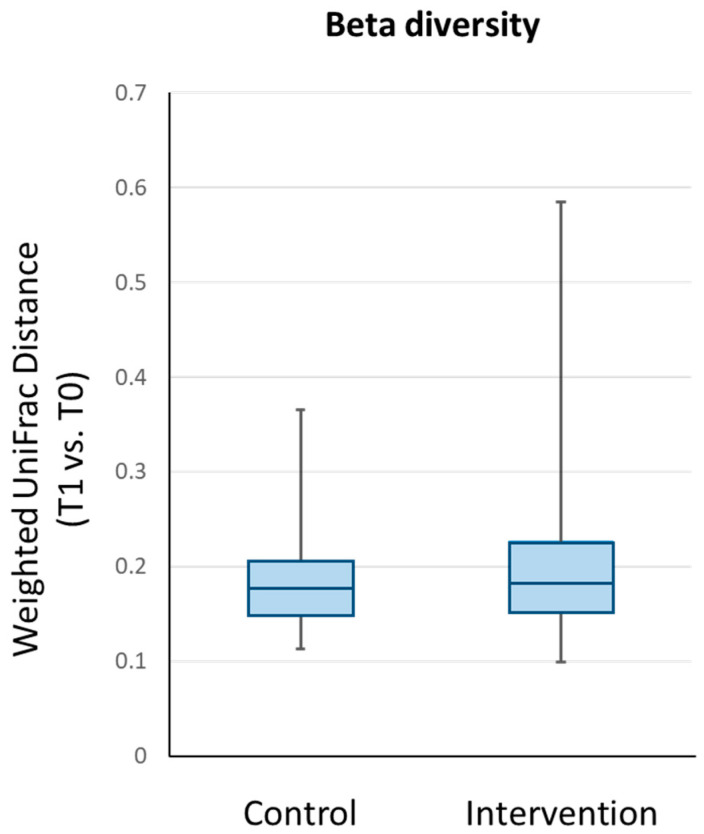
Beta diversity analysis of the participants’ intestinal microbiota using the phylogenetic-based weighted UniFrac distance. The box and whisker plots show the median, lower quartile, upper quartile, minimum value and maximum value of the within-subject pairwise distances between T1 and T0 for each group.

Figure 4 shows a graphical representation of the changes in beta diversity, which depicts the temporal changes in the overall microbial composition between T0 and T1 for the control and intervention groups. No significant between-group differences were observed for the changes in weighted UniFrac distance values (unadjusted *p* = 0.709 and sex-adjusted *p* = 0.731; *p* values were determined after log transformation of the weighted UniFrac distance).

Overall, the 24-week rice bran dietary intervention exhibited no effect on the richness and evenness of the bacterial species in the intestinal microbiota of the participants, as well as the temporal change in the overall composition of the intestinal microbiota of the participants.

### 3.3. Compliance Rate

The participants’ compliance was overall comparable between the intervention and control groups. The mean compliance rate of the intervention participants was 91.3% (range: 50–100%), and 52.6% of subjects exhibited 100% compliance. The mean compliance rate of the control participants was 96.2% (range: 57.1–100%), and 70% of subjects exhibited 100% compliance. The primary reason for the participants’ inability to exhibit 100% compliance was that they occasionally forgot to consume the rice products as prescribed. Notably, however, one participant in the intervention group discontinued the consumption of rice bran because the participant received a diagnosis of hyperkalaemia during the intervention and was concerned that rice bran intake could exacerbate the condition.

### 3.4. Adverse Events Caused by Rice Bran Intake

Adverse events associated with rice bran intake were assessed via records of any abdominal discomfort and pain among the participants in the intervention group during the 24-week intervention. The frequency and prevalence of these events among the intervention participants was compared to that among the control counterparts, to determine whether there were any inter-group differences. No abdominal discomfort or pain or any other health problems during the intervention were reported by 52.6% and 55.0% of the intervention and control participants, respectively. None of the participants withdrew from the study due to adverse events. The intervention participants who experienced abdominal discomfort or pain had such symptoms for no more than eight days during the intervention. Two subjects specified that their abdominal discomfort was mild. One participant reported having experienced stomach pain for one day, about 16 weeks after commencing rice bran consumption, but did not specify the intensity of this pain. Another participant reported symptoms such as diarrhoea and constipation, which occurred on seven days and six days, respectively, during the intervention. The control participants reported that abdominal symptoms occurred on fewer than 10 days during the intervention, and one participant reported stomach pain on four days and enterocolitis on one day.

The lack of an observable difference in the prevalence and frequency of abdominal symptoms between the intervention and control participants indicates that long-term consumption of rice bran is unlikely to have any adverse effects.

## 4. Discussion

To our knowledge, our study was the first to examine the effects of a long-term (24 weeks) rice bran dietary intervention on the intestinal microbiota of Chinese subjects. The data demonstrate that the implementation of such a long-term intervention was feasible, as indicated by the high retention rate and participants’ compliance rate, and the absence of severe adverse events during the 24-week intervention. The metagenomic data also suggest that long-term rice bran consumption may promote intestinal health by increasing the intestinal abundance of *Lactobacillus*, and possibly by enhancing that of *Bifidobacteria* and the F/B ratio of the intestinal microbiota.

Studies [15,16] have demonstrated that a 4-week rice bran dietary intervention enhanced the intestinal abundance of certain health-promoting bacteria, including *Lactobacillus*, *Ruminococcus* and *Bifidobacteria*. Our findings concur with these studies, and further show that a long-term rice bran dietary intervention may have a similar effect, by increasing abundance of the intestine by certain health-promoting bacteria, notably *Lactobacillus*. The intervention also tended to increase the intestinal abundance of *Bifidobacteria*. Moreover, our long-term rice bran dietary intervention appeared to have no effect on the intestinal abundance of pathogenic bacteria, such as *Fusobacteria*. These findings therefore provide further evidence that a long-term rice bran intervention could also induce health-promoting modification of the composition of intestinal microbiota. Furthermore, the data suggest that it is possible that rice bran exhibits its health-promoting effects primarily by enhancing the intestinal abundance of health-promoting bacteria, rather than by decreasing the abundance of pathogenic bacteria. Nevertheless, further larger-scale trials are needed to confirm this possibility.

Another major finding of this study was the difference in the F/B ratio between the intervention and control participants at T1. It has been suggested that the F/B ratio is modified in colon tumorigenesis, and a reduced F/B ratio has been found in patients with CRC [10,11]. Consistent with these findings, patients with inflammatory bowel disease, a disease shown to exhibit a positive association with the risk of CRC [27], were reported to have a lower F/B ratio than individuals without disease [28]. Here, we report that the intervention participants exhibited an increase in the F/B ratio, whilst the control counterparts reported a decrease, although the extent of these changes was small, and no significant inter-group difference was observed. This finding suggests that rice bran consumption could potentially help to reduce the risk of CRC by modifying the F/B ratio of the intestinal microbiota. The increase in the F/B ratio is likely to be caused primarily by the enrichment of *Firmicutes* in the intestine, as indicated by the significant inter-group difference in the change in its absolute abundance values from T0 to T1 (Table 4). Notably, these findings regarding the effects of rice bran consumption on the F/B ratio contradict those of our previous pilot rice bran intervention study in healthy adults [17]. This discrepancy could be explained by differences in the designs of the studies and the duration of the rice bran dietary intervention that was implemented. It is possible that rice bran consumption could lead to a short-term decrease in the F/B ratio, whilst an increase could be observed over the long term. Further studies are needed to establish whether there are temporal differences in the effects of rice bran consumption on the F/B ratio of the intestinal microbiota.

Interestingly, our data show that rice bran consumption did not lead to changes in the bacterial diversity of the participants’ intestinal microbiota, in terms of the richness and evenness of its bacterial species. This finding contradicts that of Sheflin et al. [16], who showed that 4 weeks of rice bran intake led to a significant enhancement in the number of observed species and the chao-1 index of the intestinal microbiota of patients treated for CRC. This discrepancy may be due to differences in the subjects’ ethnicity and health status between our study and their study, but it is also possible that rice bran intake may only have a short-term positive effect on the bacterial diversity of intestinal microbiota. Indeed, it has been suggested that changes in diet can only induce short-term modifications of the intestinal microbiota, and an answer to the question of whether an extended period of dietary modification can induce long-term changes in the intestinal microbiota remains elusive [29]. It is possible that whilst rice bran consumption may lead to a transient increase in bacterial diversity in the intestinal microbiota, this effect cannot be sustained over the long term. Since previous studies of rice bran dietary interventions did not involve long-term assessment of the participants’ outcomes, studies that include longer-term follow-up outcome assessments are required to determine whether the beneficial effects of short-term rice bran dietary interventions on intestinal microbiota can be sustained. Alternatively, further long-term rice bran dietary intervention studies, which include multiple follow-up outcome assessments throughout the intervention, will help to identify any short- and long-term effects of such interventions on the composition of the participants’ intestinal microbiota.

Moreover, we noted no observable difference in the occurrence of adverse effects between the two groups, suggesting that rice bran consumption would not elicit any major safety issues. Given that the observed changes in the composition of the subjects’ intestinal microbiota caused by our intervention are relatively minor, it is likely that the rice bran used in this intervention was fermentation-resistant.

### Limitations

This study has several limitations. First, the sample size in this pilot study was small and was unlikely to provide the statistical power required to draw firm conclusions regarding the effects of the intervention on the intestinal microbiota. Indeed, based on a power analysis using GPower 3.1 software, given a sample size of 20 participants in each of two parallel groups, this study has only 46%, 34%, and 23% statistical power to detect outcomes with effect sizes of 0.6, 0.5, and 0.4, respectively, at a two-sided 5% level of significance. Second, the study only involved outcome measurements at baseline and after the intervention, which limited the capacity to assess the longitudinal changes in the outcomes throughout the 24-week intervention. Third, the sample comprised Chinese subjects only, and the findings are unlikely to be generalisable to subjects of other ethnicities due to the known differences in the composition of the intestinal microbiota among individuals of various ethnicities. Fourth, although the mean compliance rate was ≥91.3%, only 53% of the participants in our study exhibited 100% compliance with the intervention. Therefore, the results may not truly reflect the effects of rice bran consumption on the subjects’ intestinal microbiota. Fifth, data on the participants’ BMI were not collected at post-intervention, a process that would have enabled the assessment of any significant changes in BMI among the participants throughout the intervention. As BMI has been suggested to influence the composition of the intestinal microbiota [30], we cannot ascertain whether the observed changes in the intestinal microbiota of the participants in this study were due to the effect of rice bran intake or changes in BMI. Sixth, the results of this study may be confounded by other unobserved nutrients, such as fats. These limitations warrant caution in the interpretation of these findings. Further studies are required to establish the beneficial effects of long-term rice bran consumption on intestinal health. These must be adequately powered and comprise a longer-term rice bran dietary intervention, with multiple follow-up assessments of both the outcomes of interest and potential confounders throughout the intervention.

## 5. Conclusions

Our study is the first to show that the implementation of a long-term (24-week) rice bran dietary intervention in Chinese subjects with a high risk of CRC is feasible, and no serious adverse effects of rice bran consumption were reported during the study. The study findings also provide further evidence that a long-term rice bran dietary intervention may promote intestinal health in such individuals by enhancing intestinal abundance of health-promoting bacterial genera such as *Lactobacillus*, and by increasing the F/B ratio, although no effect on bacterial diversity was observed. The intervention also caused a significant increase in intestinal abundance of *Firmicutes*, and tended to enhance the intestinal abundance of *Bifidobacteria, Prevotella_9* and bacteria belonging to the order of *Lactobacillales* and the class of *Bacilli*. A larger-scale study involving a longer duration of rice bran consumption and the use of multiple follow-up outcome assessments would help to establish the real effects of long-term rice bran consumption on intestinal health. Such a study would also provide insights into the potential of rice bran consumption to effectively promote intestinal health via health-promoting modification to the intestinal microbiota of healthy adults, especially those at a high risk of developing CRC. Promoting intestinal health in this manner may help reduce the risk of CRC in these individuals.

## Figures and Tables

**Table 1:** Nutrition information of the rice bran and rice powder used in this study.

Nutrients	Rice Bran (per 30 g Serving)	Rice Powder (per 30 g Serving)
Calories	111 kcal	120 kcal
Protein	3.2 g	2.0 g
Total fats	2.1 g	1.0 g
Saturated fats	0.4 g	0.0 g
Trans fats	0.0 g	0.0 g
Total carbohydrates	19.7 g	24.0 g
Of which sugars	1.0 g	<2.0 g
Dietary fibre	3.1 g	0.0 g
Sodium	1.2 mg	10 mg

**Table 2:** Characteristics of the study participants (*n* = 40).

Characteristics	Control (*n* = 20)	Intervention (*n* = 20)	*p*-Value
Age (years) ^†^	65.9 (5.4)	65.3 (4.7)	0.663 ^a^
Body mass index (kg/m^2^) ^†^	24.0 (2.6)	24.0 (2.7)	0.961 ^a^
Sex			
Male	10 (50.0%)	17 (85.0%)	0.018 ^b^
Female	10 (50.0%)	3 (15.0%)	
Educational level			
No formal education/primary	4 (20.0%)	6 (30.0%)	0.693 ^b^
Secondary	9 (45.0%)	9 (45.0%)	
Post-secondary or above	7 (35.0%)	5 (25.0%)	
Marital status			
Married	14 (70.0%)	18 (90.0%)	0.235 ^c^
Single/divorced/widowed	6 (30.0%)	2 (10.0%)	
Employment status			
Employed	6 (30.0%)	9 (45.0%)	0.327 ^b^
Unemployed/retired	14 (70.0%)	11 (55.0%)	
Current smoker			
No	18 (90.0%)	13 (68.4%)	0.127 ^c^
			
Yes	2 (10.0%)	6 (31.6%)	
Regular alcohol drinker			
No	17 (85.0%)	13 (68.4%)	0.273 ^c^
Yes	3 (15.0%)	6 (31.6%)	
Average daily fruit intake			
0–1 portion	12 (60.0%)	13 (65.0%)	0.744 ^b^
≥2 portions	8 (40.0%)	7 (35.0%)	
Average daily vegetables intake			
0–1 portion	10 (50.0%)	12 (60.0%)	0.525 ^b^
≥2 portions	10 (50.0%)	8 (40.0%)	
Average daily intake of meat, fish, egg and alternatives			
0–4 tael	12 (60.0%)	14 (70.0%)	0.507 ^b^
≥5 tael	8 (40.0%)	6 (30.0%)	
Regular meal time			
Yes	14 (70.0%)	13 (65.0%)	0.736 ^b^
No	6 (30.0%)	7 (35.0%)	

Data marked with ^†^ are presented as mean (standard deviation), all others are presented as frequency (%). ^a^ Independent *t*-test; ^b^ Pearson chi-square test; ^c^ Fisher’s exact test.

**Table 3:** Absolute abundance values indicating the faecal abundance of bacteria in various taxonomic levels at T0 and T1 among the control and intervention participants.

	Control Group (*n* = 20)		Intervention Group (*n* = 19)	
	T0 (Baseline)	T1 (24 Weeks)	T0 (Baseline)	T1 (24 Weeks)
Absolute abundance (number of reads)				
Phylum				
Firmicutes	19,211 (14,696, 21,670)	17,224 (14,512, 19,996)	15,375 (12,747, 16,761)	16,209 (13,531, 18,752)
Bacteroidetes	16,396 (15,074, 19,594)	17,674 (14,340, 20,421)	18,987 (14,466, 22,206)	19,812 (17,450, 22,988)
Proteobacteria	2703 (1904, 3589)	3568 (2386, 5157)	2526 (1926, 5051)	3954 (2040, 6645)
Actinobacteria	229 (153, 560)	366 (182, 1018)	199 (141, 307)	876 (358, 1336)
Fusobacteria	61 (33, 200)	52 (15, 136)	99 (23, 953)	54 (24, 1613)
Firmicutes/Bacteroidetes ratio	1.190 (0.866, 1.588)	1.018 (0.816, 1.239)	0.794 (0.639, 0.979)	0.775 (0.656, 0.989)
Class				
Bacteroidia	16,396 (15,074, 19,594)	17,674 (14,336, 20,420)	18,987 (14,466, 22,206)	19,801 (17,450, 22,988)
Clostridia	16,714 (12,641, 20,710)	15,230 (12,924, 18,568)	12,133 (10,841, 15,110)	13,532 (10,966, 15,929)
Negativicutes	1190 (602, 1510)	1489 (946, 1866)	834 (634, 1415)	1652 (971, 2117)
Bacilli	82 (40, 292)	79 (28, 134)	108 (43, 357)	148 (95, 196)
Gammaproteobacteria	2278 (1298, 2863)	2436 (1836, 4682)	2254 (1602, 4908)	2879 (1976, 6249)
Order				
Bacteroidales	16,394 (15,073, 19,583)	17,674 (14,230, 20,346)	18,986 (14,448, 22,206)	19,699 (17,396, 22,988)
Clostridiales	16,711 (12,640, 20,708)	15,228 (12,924, 18,567)	12,130 (10,841, 15,110)	13,531 (10,966, 15,929)
Selenomonadales	1190 (602, 1510)	1489 (946, 1866)	834 (634, 1415)	1652 (971, 2117)
Lactobacillales	38 (24, 74)	36 (26, 76)	36 (19, 53)	73 (25, 99)
Enterobacteriales	423 (226, 1190)	976 (557, 3221)	337 (117, 965)	454 (216, 865)
Family				
Bacteroidaceae	13,433 (8114, 16,306)	12,500 (8170, 17,482)	10,808 (5397, 15,592)	12,226 (4698, 15,899)
Prevotellaceae	602 (366, 1484)	548 (456, 1750)	1021 (548, 11,807)	971 (553, 10,564)
Clostridiaceae_1	50 (30, 83)	22 (12, 36)	43 (18, 136)	33 (15, 59)
Lachnospiraceae	6294 (5660, 8950)	6947 (5388, 9041)	6937 (5385, 8577)	6638 (4647, 8269)
Veillonellaceae	694 (260, 1266)	812 (340, 1474)	306 (168, 1075)	542 (219, 1874)
Genus				
Bacteroides	13,433 (8114, 16,306)	12,500 (8170, 17,482)	10,808 (5397, 15,592)	12,226 (4698, 15,899)
Prevotella_9	400 (227, 1000)	441 (304, 1031)	472 (369, 9805)	491 (310, 9629)
Clostridium_sensu_stricto_1	50 (30, 83)	22 (12, 34)	43 (18, 136)	32 (15, 51)
Veillonella	10 (7, 30)	41 (14, 95)	11 (3, 35)	18 (12, 91)
Lactobacillus	0 (0, 18)	1 (0, 18)	0 (0, 31)	36 (0, 62)
Bifidobacterium	122 (70, 198)	129 (63, 197)	63 (30, 124)	162 (93, 395)
Faecalibacterium	2734 (1470, 3684)	2270 (1780, 3014)	2410 (1307, 3467)	2326 (728, 3324)
Escherichia-Shigella	206 (122, 416)	421 (273, 1354)	83 (64, 283)	206 (133, 322)
Blautia	142 (102, 192)	108 (87, 204)	133 (100, 202)	113 (74, 199)
Roseburia	498 (290, 1160)	514 (334, 926)	614 (292, 941)	605 (371, 1638)
Fusobacterium	52 (30, 200)	52 (15, 136)	99 (17, 953)	54 (24, 1613)
Ruminococcus_1	224 (133, 529)	216 (161, 346)	183 (78, 345)	174 (84, 262)
Ruminococcus_2	384 (238, 619)	508 (282, 762)	351 (145, 631)	506 (197, 709)
Species				
Faecalibacterium prausnitzii	5 (2, 8)	6 (3, 10)	2 (0, 6)	4 (1, 7)
Bifidobacterium longum	18 (5, 32)	19 (12, 36)	8 (3, 29)	20 (12, 38)
Clostridium butyricum	12 (0, 42)	0 (0, 2)	0 (0, 38)	0 (0, 3)
Bacteroides fragilis	45 (17, 192)	68 (26, 223)	42 (15, 102)	68 (23, 126)

Data are presented as median (inter-quartile range).

**Table 4:** Changes in the absolute abundance values indicating the faecal abundance of bacteria in various taxonomic levels between T0 and T1 among the control and intervention participants.

	Change from T0 to T1 (T1–T0)			
	Control Group (*n* = 20)	Intervention Group (*n* = 19)	*p*-Value (Unadjusted)	*p*-Value (Sex-Adjusted)
Absolute abundance (number of reads)				
Phylum				
Firmicutes	−2160 (−4778, 1964)	1473 (−1351, 5285)	0.022	0.018
Bacteroidetes	899 (−2052, 4177)	−48 (−3987, 5346)	0.564	0.525
Proteobacteria	1046 (−512, 2320)	682 (−1313, 2184)	0.797	0.304
Actinobacteria #	98 (−82, 698)	697 (72, 1168)	0.143	0.129
Fusobacteria #	−2 (−40, 30)	−13 (−46, 100)	0.362	0.382
Firmicutes/Bacteroidetes ratio #	−0.131 (−0.447, 0.017)	0.059 (−0.152, 0.241)	0.079	0.054
Class				
Bacteroidia	897 (−2052, 4177)	−48 (−3987, 5335)	0.564	0.525
Clostridia	−2700 (−5066, 2203)	752 (−2631, 2811)	0.249	0.262
Negativicutes #	310 (10, 684)	759 (14, 1474)	0.266	0.289
Bacilli #	−28 (−212, 60)	31 (−199, 111)	0.153	0.179
Gammaproteobacteria	984 (−462, 1728)	469 (−1286, 2175)	0.810	0.662
Order				
Bacteroidales	794 (−2063, 4156)	−36 (−3989, 5251)	0.561	0.522
Clostridiales	−2696 (−5065, 2202)	752 (−2628, 2811)	0.248	0.262
Selenomonadales #	310 (10, 684)	759 (14, 1474)	0.266	0.289
Lactobacillales #	−2 (−22, 24)	38 (−9, 65)	0.096	0.146
Enterobacteriales #	234 (−39, 1408)	42 (−125, 380)	0.563	0.754
Family				
Bacteroidaceae	480 (−2570, 2966)	553 (−2703, 2882)	0.942	0.660
Prevotellaceae	42 (−288, 788)	−389 (−2707, 485)	0.186	0.281
Clostridiaceae_1	−26 (−48, −3)	−12 (−100, 34)	0.323	0.457
Lachnospiraceae	−88 (−2448, 1773)	−341 (−1757, 1461)	0.744	0.641
Veillonellaceae #	154 (−170, 402)	121 (9, 1210)	0.253	0.255
Genus				
Bacteroides	480 (−2570, 2966)	553 (−2703, 2882)	0.942	0.660
Prevotella_9	100 (−266, 536)	−159 (−362, 359)	0.117	0.160
Clostridium_sensu_stricto_1	−28 (−50, −4)	−11 (−100, 32)	0.342	0.481
Veillonella #	18 (2, 61)	10 (−2, 67)	0.841	0.831
Lactobacillus	0 (0, 4)	19 (0, 43)	0.026	0.033
Bifidobacterium #	28 (−120, 92)	91 (12, 162)	0.125	0.131
Faecalibacterium	−486 (−983, 948)	−240 (−1506, 955)	0.862	0.582
Escherichia-Shigella	190 (20, 898)	64 (−6, 225)	0.386	0.914
Blautia	−26 (−100, 64)	−5 (−99, 50)	0.527	0.611
Roseburia #	38 (−196, 420)	143 (−551, 421)	0.376	0.656
Fusobacterium	2 (−40, 36)	−3 (−46, 120)	0.815	0.840
Ruminococcus_1 #	7 (−208, 108)	29 (−148, 153)	0.879	0.962
Ruminococcus_2 #	111 (−62, 536)	−16 (−215, 347)	0.820	0.543
Species				
Faecalibacterium prausnitzii *	2 (−4, 6)	1 (0, 4)	0.841	0.682
Bifidobacterium longum *	10 (−14, 22)	12 (−3, 27)	0.365	0.261
Clostridium butyricum *	−12 (−39, 0)	0 (−36, 0)	0.571	0.660
Bacteroides fragilis *	44 (−16, 84)	16 (−62, 69)	0.476	0.647

Data are presented as median (inter-quartile range) and compared between groups by using linear regression. # log-transformed before being compared between the intervention and control groups using linear regression. * square root transformed before being compared between the intervention and control groups using linear regression.

## Data Availability

The data that support the findings of this study are available on request from the corresponding author, W.K.W.S.

## Supplementary Materials

The following are available online at https://www.mdpi.com/2072-6643/13/2/526/s1, Figure S1: Analysis of the intestinal abundance of various phyla among the participants in a heat map. (T0—C: Control group at T0; T0—I: Intervention group at T0; T1—C: Control group at T1; T1—I: Intervention group at T1).
