# Supplementary figures and images for: Effects of a Rice Bran Dietary Intervention on the Composition of the Intestinal Microbiota of Adults with a High Risk of Colorectal Cancer: A Pilot Randomised-Controlled Trial
Source: Nutrients. 2021 Feb 6;13(2):526. doi: 10.3390/nu13020526 (PMC7915415; doi:10.3390/nu13020526)

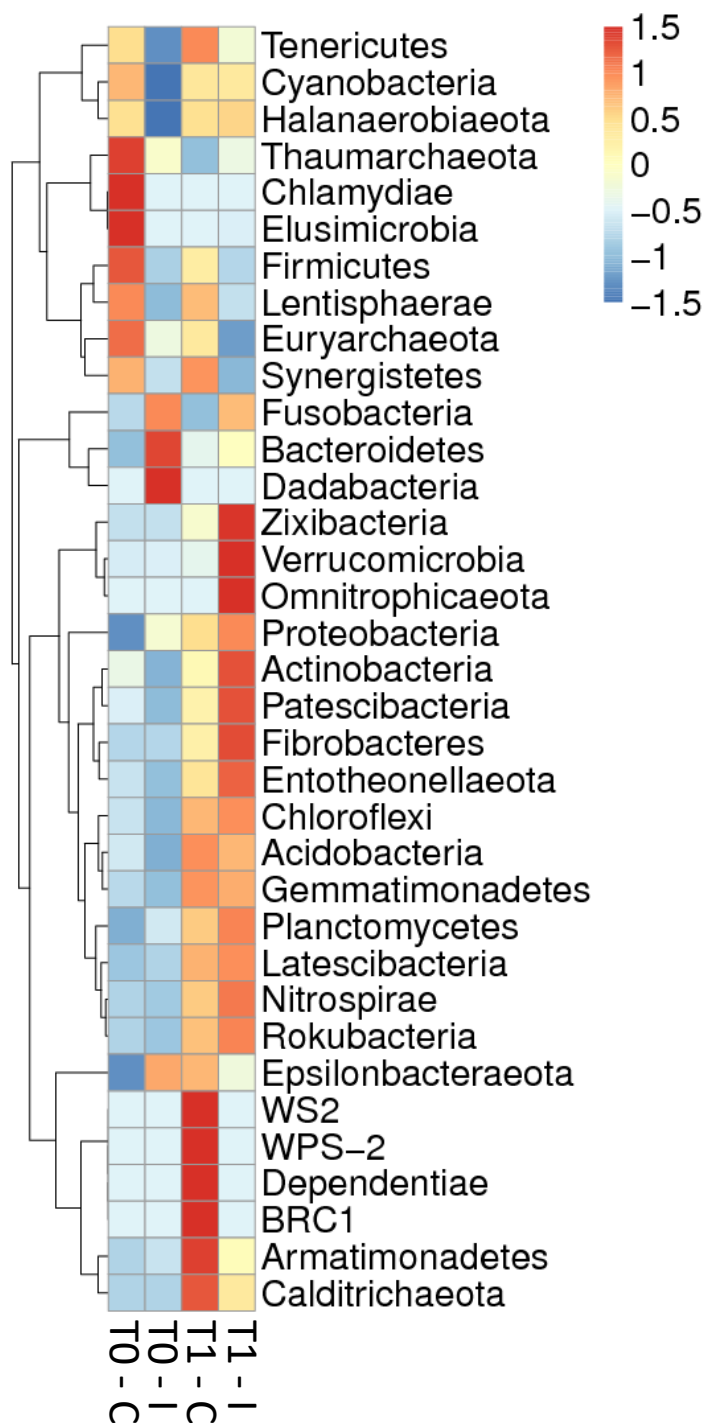

Supplement: Supplementary file 1 [file nutrients-13-00526-s001.pdf]
